# If looks could kill: Fungal macroscopic morphology and virulence

**DOI:** 10.1371/journal.ppat.1008612

**Published:** 2020-06-18

**Authors:** Caitlin H. Kowalski, Robert A. Cramer

**Affiliations:** Department of Microbiology and Immunology, Geisel School of Medicine at Dartmouth, Hanover, New Hampshire, United States of America; University of Maryland, Baltimore, UNITED STATES

## Overview

The complexity and diversity of microbial colony morphologies have contributed to the identification of pathogenic microbes for decades. Even as biomarker-based approaches are adopted for diagnosis of fungal infections, culture-based methods remain valuable for the identification of specific etiological agents and determination of antifungal susceptibility [1]. Thus, when obtainable, infectious organisms are observed as macroscopic colony biofilms in clinical settings. We define macroscopic morphologies (a strain’s morphotype) as the collective phenotypes of form and/or structure attributed to a group of organisms of the same species within a defined area such as a colony. Clinical isolates of *Aspergillus fumigatus*, *Cryptococcus neoformans*, and *Candida* spp. have been reported for decades to be morphologically variable within and between patients [2–4]. Similarly, diverse population-level macroscopic morphologies of bacterial pathogens have been observed in clinical samples [5,6]. A key question is whether observed microbial morphotypes tell us anything about their virulence. Below, we discuss the intrinsic and extrinsic factors that contribute to macroscopic morphological variation in fungi and bacteria. We then discuss fungal and bacterial examples linking macroscopic morphology with virulence and the challenges faced with studying this relationship. Finally, we discuss the importance of investigating recurring and distinct fungal macroscopic morphologies for furthering our understanding of host–fungal interactions.

## What features of population-level growth contribute to macroscopic morphologies?

Microbial populations are most often observed as macroscopic colonies with defined morphologies on a solid surface. Underlying features of macroscopic microbial colonies include the production and secretion of extracellular matrix (ECM) [7], quorum sensing [8], surface appendages [9], pigmentation [10,11], and cellular morphology [12]. Importantly, these features often, but not always, impact a given strain’s macroscopic morphology. For example, quorum sensing in *Pseudomonas aeruginosa* regulates the production of phenazines that, under permissive conditions, contribute to colony pigmentation and topography [8]. The production of the long filamentous pilus is reflected in the colony morphology of *Neisseria gonorrhoeae*, in which colonies without a pilus are flat and lack a distinct colony edge [9]. However, while some features may broadly impact microbial colony morphologies, others are species-specific. For example, the ECM of *Vibrio cholerae* is necessary for the rugose colony morphology [7], but in the pathogenic filamentous fungus *A*. *fumigatus*, loss of the primary ECM component galactosaminogalactan does not impact colony morphology [13].

In yeast, the ECM influences fungal colony topography such as in *Candida albicans*, *C*. *neoformans*, and *Saccharomyces cerevisiae* [14]. The mucoid colony morphotype of *C*. *neoformans* coincides with increased thickness of the surface capsule, a key virulence attribute of this yeast [15]. In addition to ECM, colony morphotypes of *Candida* spp., *C*. *neoformans*, and *S*. *cerevisiae* are impacted by the presence of various cell morphologies, including yeast, pseudophyphae, and hyphae [14]. Yeast cellular-morphology–based colonies are smooth and structureless, while colonies that contain pseudohyphae or hyphae are wrinkled or fluffy and highly structured [14]. Colonies of *A*. *fumigatus* reflect cell morphology changes as well, in which vegetative hyphae become conidiophores. These specialized structures produce pigmented conidia, and the pattern of conidiation is a prominent and quantifiable feature of *A*. *fumigatus* colony morphology that reflects changes in cellular physiology [13]. Notably, many of these macroscopic colony features are involved in pathogenesis and virulence, discussed further below.

## What genetic factors contribute to features of macroscopic morphologies?

Example genetic mechanisms that impact macroscopic morphology include loss or gain of function alleles, reversible phenotype switching, phase variation, and aneuploidy. Among bacteria, a few of the best-characterized molecular mechanisms underlying macroscopic morphological variation include loss of function alleles of the quorum sensing transcriptional regulator *lasR* of *P*. *aeruginosa* [8], reversible induction of the *vps* (vibrio polysaccharide) operon of *V*. *cholerae* [7], and phase variation of pilin proteins of *N*. *gonorrhoeae* [9].

Among fungi, the molecular mechanisms underlying macroscopic morphologies are also diverse. *C*. *albicans* undergoes phenotype switching in two dimensions at the cellular level, which impacts population-level morphologies. The yeast-to-hyphae transition is one example, induced by transcriptional regulators *EFG1*, *BRG1*, and *NDT80*, among others [12]. Secondly, white–gray–opaque phenotype switching alters the yeast cellular morphology and colony morphotypes as a result of transcriptional rewiring [14]. *C*. *albicans* also generates macroscopic morphotype variation as a result of chromosome instabilities and aneuploidies [16]. Ploidy has also been observed to impact colony morphotypes of *S*. *cerevisiae*, in which isogenic haploid and diploid colonies are morphologically distinct [17]. For *C*. *neoformans*, chromosome instabilities do not consistently correlate with variation in colony morphology, and the molecular mechanism(s) facilitating phenotypic switching in this yeast remain largely unknown [15].

Among the human pathogenic molds, molecular mechanisms driving colony morphology are beginning to emerge. For *A*. *fumigatus*, reverse genetic approaches have identified genes that simultaneously impact colony morphology and cell wall biosynthesis. These include the polysaccharide synthase *cpsA* that contributes to cell wall integrity, the chitin synthases *chsC* and *chsG*, and the β-1,3-glucan glycosyltransferase *gel2* [18]. Additionally, the induction of a subtelomeric gene cluster through a hyperactive allele of its putative regulator *hrmA* modifies the macroscopic morphology of *A*. *fumigatus* colonies [13]. As mentioned above, developmental transcriptional rewiring also impacts colony morphology of *A*. *fumigatus*. For example, transcriptional regulators of asexual development such as *stuA*, *brlA*, and others are molecular contributors to macroscopic morphology [19]. Additionally, mycovirus infection has been observed to modulate *A*. *fumigatus* colony morphology; however, the mechanisms remain unknown [20]. Which of these molecular mechanisms contribute to the natural macroscopic morphotype variation among clinical isolates of *A*. *fumigatus* and other fungi remains to be discovered.

Taken together, it seems clear that the genetic factors that influence macroscopic morphologies are equally as diverse as the underlying physiological pathways. The question is thus raised: is the altered colony morphology responsible for changes in virulence, or is it other functions of the underlying perturbed genetic factor? The answer may not be mutually exclusive, and this question represents a challenge when understanding macroscopic morphology and its potential association with virulence. In either case, the change in macroscopic colony morphology may be indicative that a key genetic factor associated with virulence has been changed in the microbe under study. Thus, defining the genetic mechanisms driving morphological change should be an important goal for future research.

## What environmental factors influence macroscopic morphologies?

Another complex question to appreciate in order to understand the relationship between macroscopic morphology and virulence is the impact of the environment. Fungal macroscopic morphologies are often highly dependent on environmental conditions. Extrinsic factors such as carbon and nitrogen sources and abundance, oxygen availability, agar concentration, and proximity to other microorganisms are known to impact morphology. Are the culture conditions in which the distinct morphotype is observed at all similar to the in vivo infection microenvironment? Understanding the environmental impact on macroscopic morphology may yield important insights into underlying pathogenesis associated mechanisms.

**Fig 1:**
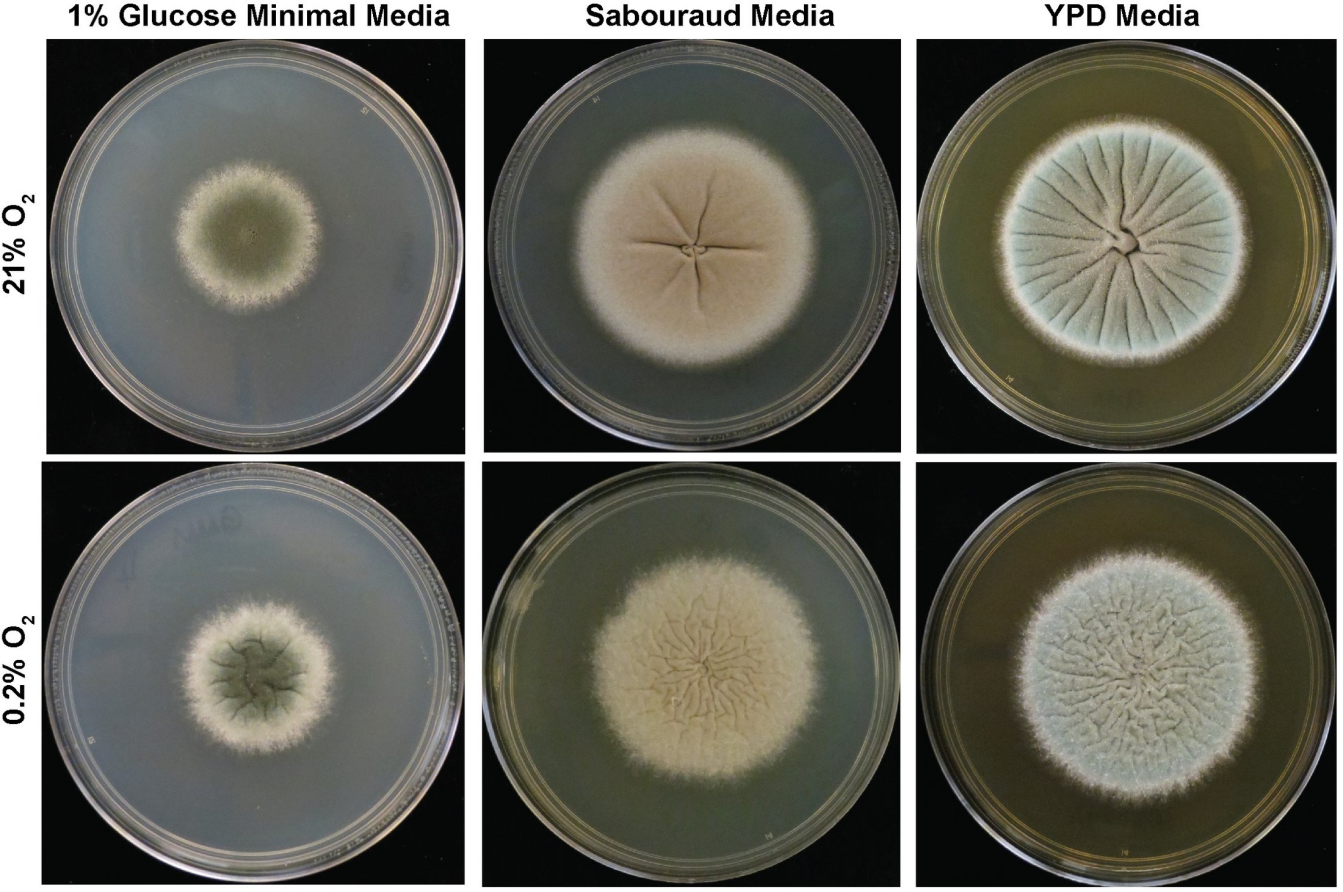
*A*. *fumigatus* strain AF293 cultured on minimal and complex media for 72 hours. Representative images of 1,000 AF293 conidia spot-inoculated on 1% glucose minimal media, Sabouraud complex media, or YPD complex media. For each media type, hypoxia exacerbates colony topographical features. Growth on complex media sources results in colony furrows, whereas growth on minimal media does not under the same atmospheric conditions. YPD, yeast peptone dextrose.

For example, extrinsic inducers of pseudohyphal or hyphal growth for yeast such as *C*. *albicans*, *C*. *neoformans*, and *S*. *cerevisiae* impact macroscopic morphotypes [14]. For *S*. *cerevisiae*, glucose starvation in the presence of a rich nitrogen source results in highly structured, wrinkled colonies characteristic of pseudohyphae formation [17]. For *C*. *albicans*, growth in low oxygen is one of several inducers of hyphal growth and results in a switch from smooth colonies at ambient oxygen to wrinkled, filamentous colonies at 1% oxygen [21]. Biotic extrinsic factors also impact *C*. *albicans* colony morphotypes. *C*. *albicans* colonies grown in close proximity to *P*. *aeruginosa* under hyphae-inducing conditions transition from a wrinkled to smooth morphotype [22]. For *Cryptococcus* spp., phenotype switching occurs stochastically at a basal rate in vitro, but also occurs inside a mammalian host [14]. *A*. *fumigatus* colony morphotypes are similarly impacted by diverse abiotic factors. The reference strain AF293 of *A*. *fumigatus* forms phenotypically distinct morphotypes on different media at ambient oxygen (Fig 1). In response to low oxygen on minimal media, *A*. *fumigatus* strains develop an array of morphotypes [23]. For the majority of strains, low oxygen induces colony furrows (Fig 1), but in others, such as the reference strain CEA10, a fluffy colony morphotype is generated [23]. Another clinically relevant environmental feature that can impact *A*. *fumigatus* and yeast morphotypes is antifungal drug treatment. For example, the echinocandin caspofungin has dose-dependent effects on *A*. *fumigatus* colony morphotypes [24]. Future research should identify other environmental factors that impact fungal morphotypes. One specific area for study that is likely to reveal novel insights is interactions with host- or other-microbial–derived metabolites at sites of colonization and infection.

## Why should we care about fungal macroscopic morphologies?

**Fig 2:**
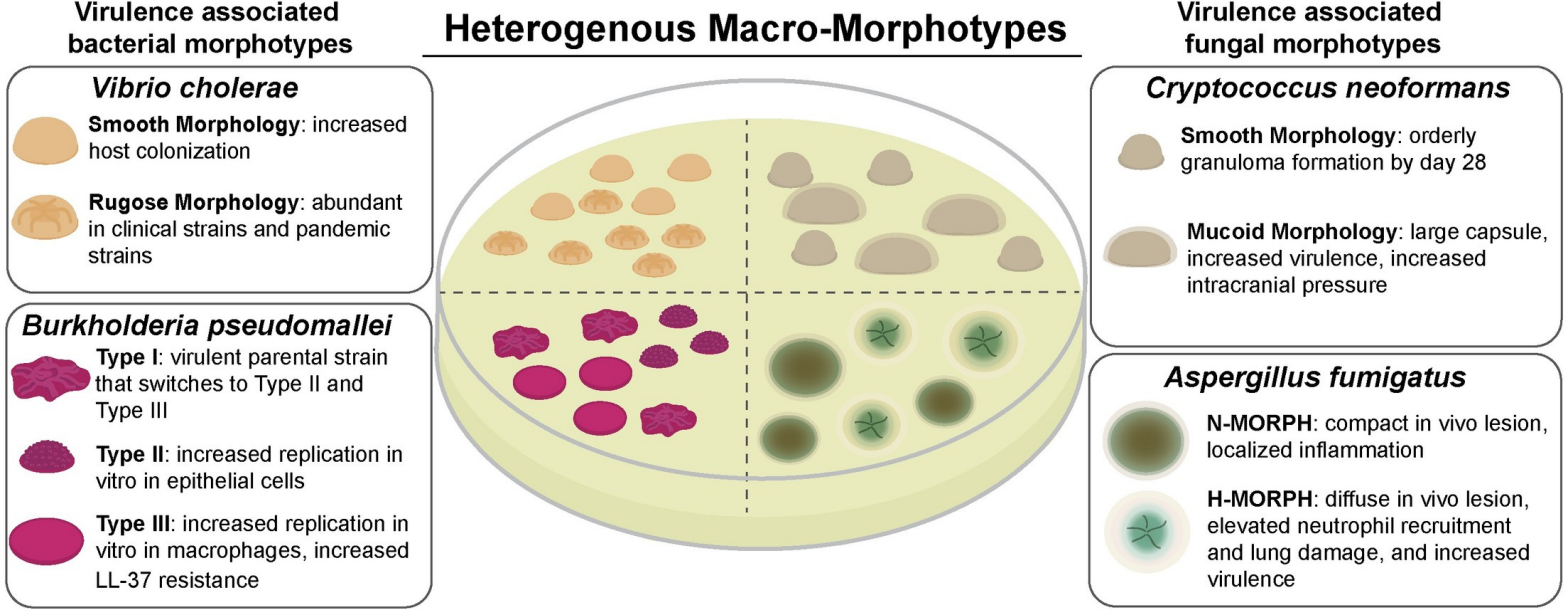
Virulence-associated macroscopic morphotypes of bacteria and fungi. Image summary of specific microbial colony morphologies associated with clinical isolation, host–pathogen interactions, and disease outcomes for bacteria (*V*. *cholerae* and *B*. *pseudomallei*) and fungi (*A*. *fumigatus* and *C*. *neoformans*) discussed in the text. H-MORPH, hypoxia-locked morphotype; LL-37, human cathelicidin family antimicrobial peptide; N-MORPH, normoxia-locked morphotype.

Distinct colony morphology variants have been associated with clinical isolates and worse clinical outcomes for a number of human bacterial and fungal pathogens. Examples of bacterial pathogens include *V*. *cholerae* [25], *Burkholderia pseudomallei* [5], nontuberculosis *Mycobacterium* spp. [26,27], *Clostridium difficile* [28], *P*. *aeruginosa* [8,29], *Staphylococcus aureus* [30,31], and *N*. *gonorrhoeae* [32] (Fig 2). Distinct bacterial and fungal colony morphotypes are also observed in clinical samples from specific body sites. In particular, this observation from clinical microbiology highlights the opportunity to define host-relevant factors that impact microbial colony biofilm morphologies and potentially virulence [5,6].

As mentioned above, for some of these bacterial species, the variation in morphotype has been directly linked to known pathways implicated in pathogenesis, such as the pilus of *N*. *gonorrhoeae* that is required for virulence [33]. In other cases, the physiology underlying an association between colony morphotypes and virulence is less clear. As an example, the etiological agent of melioidosis, *B*. *pseudomallei*, is capable of generating several distinct colony morphotypes through largely unknown mechanisms [5]. The type II and type III morphotypes, induced from type I through growth in nutrient-limited conditions for 21 days, are associated with biofilm formation and biofilm structure and altered responses to hydrogen peroxide and antimicrobial peptides, as well as differential replication within host cells [5,34,35].

Examples of the association between fungal macroscopic morphologies and virulence include the dimorphic yeast *Paracoccidioides brasiliensis* [36], the dimorphic yeast *C*. *neoformans* [37,38], and the mold *A*. *fumigatus* [13] (Fig 2). One of the *C*. *neoformans* colony morphotype variants forms a mucoid-like colony that is hypervirulent, has an enlarged polysaccharide capsule, and alters the host inflammatory response [38,39]. Perhaps a more common *C*. *neoformans* morphology is the dull or smooth colony phenotype associated with reduced or absent capsule production (for an excellent review of *Cryptococcus* morphological variants, see [15]). Importantly, both mucoid and smooth colony variants are isolated from patient samples [40,41]. A morphotype recently described for *A*. *fumigatus* has characteristics of colonies grown in hypoxia—conditions of oxygen limitation—and is referred to as a hypoxia-locked morphotype (H-MORPH) [13]. H-MORPH strains result in diffuse fungal lesions in vivo in a murine model, which is accompanied by massive inflammation and increased virulence [13]. While more H-MORPH strains need to be examined, the occurrence of the H-MORPH colony morphology in vitro may correlate with increased immunopathogenesis in vivo.

The investigation of in vivo cellular morphologies has been critical for gaining insight into pathogenic mechanisms of fungi. The yeast-to-hyphal transition is essential for virulence of *C*. *albicans*, and the generation of Titan cells of *C*. *neoformans* in vivo facilitates disease progression [42,43]. Thus, continued rigorous investigation of distinct population-level fungal morphotypes would be equally insightful. The presence of morphotypes in clinical specimens associated with specific aspects of fungal physiology such as secondary metabolite production, asexual development, or oxidative stress resistance could indicate pathways critical for fitness in vivo. Additionally, the ability to infer physiological characteristics based on colony morphology could reveal details of the infection and/or antimicrobial drug resistance. For example, the altered in vivo lesions of *A*. *fumigatus* H-MORPH colony morphotypes indicate that host morbidity is largely the result of increased inflammatory damage [13]. Similarly, constitutive induction of the asexual development program in *A*. *fumigatus* severely attenuates pathogenesis [44].

Looking forward, these observations discussed herein, when synthesized with additional clinical data, could inform new treatment strategies. The possibilities are currently limited by our understanding of the fungal or microbial physiology underlying specific morphotypes. As alluded to, an important area for future research is a better understanding of in vivo microbial morphologies. What does seem clear is that the “looks” of a fungal population should be considered important phenotypes worth reporting and investigating in further mechanistic depth. While it remains unclear whether fungal macroscopic morphology virulence associations will be the exception or the norm, the potential ability to infer pathogenesis-related phenotypes from an isolate’s morphotype is expected to continue to yield new insights into fungal biology and virulence.
